# Supplementary material for: Taqman qPCR Quantification and Fusarium Community Analysis to Evaluate Toxigenic Fungi in Cereals
Source: Toxins (Basel). 2022 Jan 6;14(1):45. doi: 10.3390/toxins14010045 (PMC8780373; doi:10.3390/toxins14010045)
Supplement: Supplementary file 1 [file toxins-14-00045-s001.zip › toxins-1524744-supplementary.pdf]

toxins

MDPI

# Supplementary Materials: Taqman qPCR Quantification and *Fusarium* Community Analysis to Evaluate Toxigenic Fungi in Cereals

**Elina Sohlberg, Vertti Virkajärvi, Päivi Parikka, Sari Rämö, Arja Laitila and Tuija Sarlin**

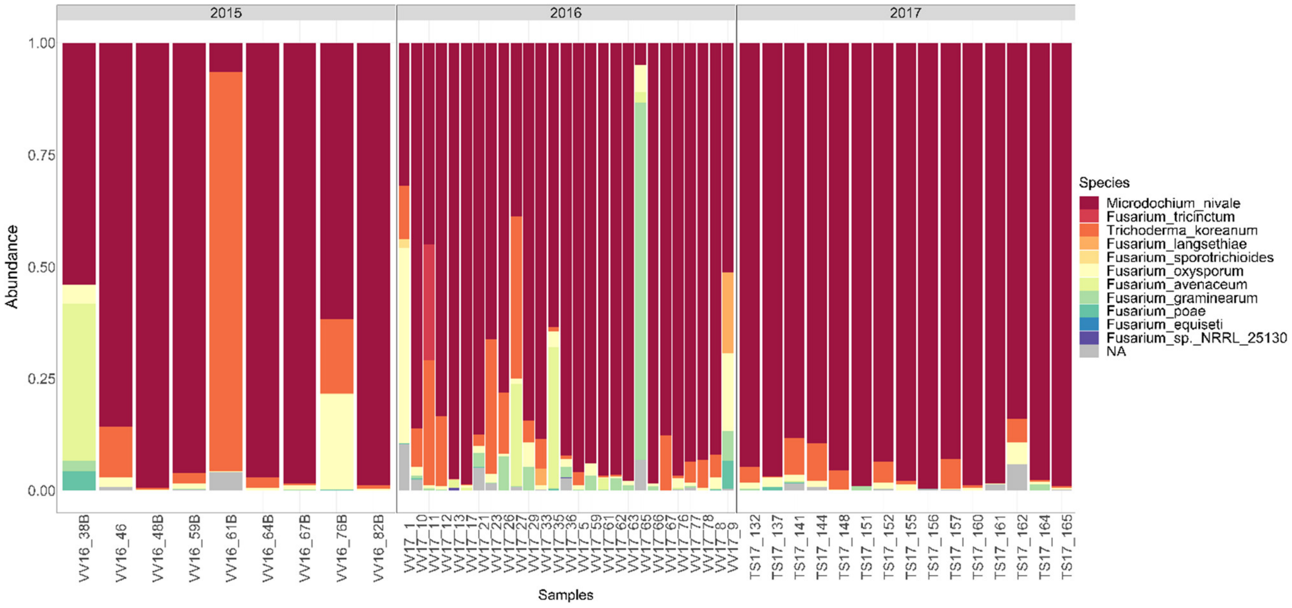


**Figure S1.** Relative abundance of all the species detected with the *Fusarium* metabarcoding method including non-*Fusarium* species in the oat samples in years 2015-2017. NA: Fusarium sp., not identified to species level.
